# Antioxidant, anti-amylase, anti-lipase, and efficiency of *Satureja* fatty acid on the anti-inflammatory parameters in lipopolysaccharide-stimulated macrophage through Nrf2/NF-kB/NADH oxidase pathway

**DOI:** 10.1038/s41598-024-63205-6

**Published:** 2024-05-31

**Authors:** Elham Obeidnejad, Gholamreza Kavoosi, Mohammad Jamal Saharkhiz

**Affiliations:** 1 Department of Biotechnology, School of Agriculture, Shiraz University, Shiraz, 7144113131 Iran; 2 Department of Horticultural Science, School of Agriculture, Shiraz University, Shiraz, Iran

**Keywords:** Herbal medicine, Aromatic plant, Lipid metabolism, Lipase, Amylase, NOX, Biochemistry, Plant sciences

## Abstract

*Satureja* is an aromatic plant that is used for flavoring, perfume, and food manufacturing due to its pleasant essential oil. Modern medicine research revealed several biological activities of *Satureja* essential oil, including antifungal, antibacterial, antiviral, antioxidant, anticancer, and anti-inflammatory. However, the functional properties of *Satureja* fatty acid have not been explored. This study examined the fatty acid profile, lipid nutritional quality, antioxidant, anti-amylase, and anti-lipase capacities of *Satureja*. The efficiency of *Satureja* fatty acid on the anti-oxidative and anti-inflammatory parameters in LPS-induced macrophage through the Nrf2/NF-kB/NADH oxidase pathway was examined. The whole lipid extract was prepared with chloroform/methanol/water solution. Fatty acids methyl ester from whole lipid extract were prepared with methanol/sulfuric acid reagent. The fatty acid profile was analyzed using gas chromatography-mass spectrometry. Total antioxidant was determined by ABTS decolorization. Lipase and amylase activities were determined by monitoring the decomposition of *p*-nitrophenyl butyrate and starch. The macrophage cell line was grown in DMEM media in the presence of fatty acid. The hydrogen peroxide production in treated cells was monitored using the FOX reagent. NADH oxidase activity was measured by monitoring NADH breakdown. The expression of NOX, NF-kB, and NRF2, were tested in the treated cells by real-time PCR. The main components of the *Satureja* fatty acid were linolenic acid (24.67–37.32%), palmitic acid (10.65–20.29%), linoleic acid (8.31–13.39%), oleic acid (4.42–14.35%), stearic acid (2.76–8.77%) and palmitoleic acid (1.77–4.95%). Given the nutritional quality, omega-3 PUFA (23.58–37.32%), SFA (21.53–26.70%), omega-6 PUFA (10.86–16.14%), omega-9 MUFA (4.42–14.35%), and omega-7 MUFA (1.77–4.95%) comprise the majority of fatty acids. *Satureja* fatty acid has a promising unsaturation index (120.77–164.27), PUFA/MUFA (2.07–6.41), hypocholesterolemic index (2.44–3.47), health-promoting index (2.03–2.42), PUFA/SFA (1.37–1.94), nutritive value index (0.53–1.71), MUFA/SFA (0.30–0.80) omega-6/omega-3 (0.34–0.65), atherogenicity index (0.41–0.49), and thrombogenicity index (0.17–0.27). *Satureja* fatty acid displayed strong antioxidant capacity (with IC_50_ ranging from 354 to 428 µg/mL), anti-lipase capacity (with IC_50_ ranging from 354 to 428 µg/mL), and anti-amylase capacity (with IC_50_ ranging from 370 to 390 µg/mL). LPS induced the expression of NOX, NRF2, and NF-kB and the synthesis of hydrogen peroxide in macrophage cells. In LPS-stimulated macrophages, *Satureja* fatty acid reduced NOX expression, hydrogen peroxide, and NF-kB expression and increased NRF2 at 0.04 mg/mL. In conclusion, *Satureja* fatty acids have potent antioxidant, anti-amylase, anti-lipase, and anti-inflammatory activities. The mechanisms in lowering oxidative stress markers depended on down-regulating superoxide-producing enzymes at gene and protein levels. *Satureja* polyunsaturated omega-3 fatty acids could be recommended for healthy products combined with dietary therapy to treat obesity, diabetes, and oxidative stress.

## Introduction

Obesity, diabetes, and inflammation are the most severe multifactorial diseases caused by hyperglycemia, hyperlipidemia, and oxidative stress. Hyperglycemia and hyperlipidemia facilitate superoxide accumulation and oxidative stress response by increasing the hexosamine pathway, polyol pathway, protein glycation, protein oxidation, and lipid oxidation^1^. The superoxide-generating enzyme (NADPH oxidase, NOX) is the most critical contributor to superoxide production in animal cells in diabetes and obesity conditions. Superoxide oxidizes various biomolecules, leading to cellular oxidative stress and cell death. Superoxide-induced cell death and disruption of the oxidants/antioxidants balance have been reported in pathological degenerative diseases such as diabetes, obesity, and inflammation^1^. The healthy body relies on the endogenous non-enzymatic (ascorbic acid, tocopherol, glutathione, carotenoid, lipoic acid) as well as enzymatic (superoxide dismutase, catalase, peroxidases) antioxidant factors to counteract against the oxidative stress impact^1^. The endogenous antioxidant mechanism is insufficient in some pathological disorders, such as metabolic syndrome. Therefore, the body needs exogenous natural antioxidants from edible plants and diet. Polyphenols, flavonoids, alkaloids, monoterpenes, monoterpenoids, and condensed tannins from medicinal plants attract growing scientific attention due to their potential beneficial effects on antioxidant potential against glucose, lipid, and protein oxidation^2^. However, only a few studies have examined the roles of omega-3 and omega-6 polyunsaturated fatty acids (PUFA) in oxidative stress, obesity, and diabetes pathogenesis.

*Satureja* is an aromatic plant commonly used as a culinary herb that is utilized in meals for flavoring, perfume, and food manufacturing due to its robust and pleasantly spicy aroma^3^. In traditional Iranian medicine, the leaves and flowers of *Satureja* are used as a stomachic, diuretic, and carminative^4^. There is also the argument of pharmacological evidence for treating cramps, muscle pains, stomach pain, indigestion, nausea, diarrhea, infectious diseases, and blood pressure, reducing inflammatory illnesses, and managing lipids and sugar in the blood using *Satureja* based on traditional usage^4^. Most research on this plant has mainly focused on the volatile oil. Carvacrol, cymene, terpinene, thujene, pinene, myrcene, thymol, linalool, and caryophyllene are the primary ingredients of *Satureja* essential oil^5^. Modern medicine and pharmacology research revealed several biological activities of *Satureja* essential oil, including antifungal^6^, antibacterial^6^, antiviral^7^, antioxidant^8^, anti-inflammatory^9^, anti-proliferative^10^, regulation of blood lipids^11^ and inhibition of lipid peroxidation^12^.

The antioxidant, anti-amylase (anti-diabetes), anti-lipase (anti-obesity), and efficiency of *Satureja* fatty acid on the anti-oxidative and anti-inflammatory parameters in lipopolysaccharide (LPS)-stimulated macrophage have not been explored. The common aspect between diabetes, obesity, and inflammation is oxidative stress. Hyperglycemia (diabetes), hyperlipidemia (obesity), and inflammation lead to the production of reactive hydrogen species (ROS) generation and oxidative stress. This study aimed to examine the antioxidant, anti-amylase, anti-lipase, and efficiency of *Satureja* polyunsaturated omega-3 fatty acid on the anti-oxidative and anti-inflammatory parameters in LPS-induced macrophage through Nrf2/NF-kB/NADH oxidase pathway. This work revealed that *Satureja* polyunsaturated omega-3 fatty acids could alleviate oxidative stress in LPS-treated cells. These data suggest that *Satureja* polyunsaturated omega-3 fatty acids could be an antioxidant therapy in oxidative stress associated with diabetes, obesity, and inflammatory responses.

## Materials and methods

### Herbal materials

*Satureja hortensis* L. leaves were taken from an experimentation field. Following identifying the plant species, the voucher specimens (Herbarium number 538F19) were added to the herbarium at Shiraz University. *Satureja bakhtiarica* Bunge (Herbarium number MPH-1577) was collected from Semirom, Esfahan (central part of Iran). Aerial parts of *Satureja sahendica* Bornm (Herbarium number: MPH-1582) and *Satureja khuzistanica* Jamzad (Herbarium number MPH-1588) from Mazhin, Lorestan, at the whole flowering stage were collected. *Satureja rechingeri* Jamzad (Herbarium number: MPH-1348) and *Satureja mutika* (Herbarium number MPH-1356) from Dehloran, Ilam (western parts of Iran) at the whole flowering stage were collected^13^. Professor Ahmad Reza Khosravi (Faculty of Science, Shiraz University, Shiraz, Iran), an experienced botanical taxonomist, recognized and confirmed this plant taxonomically. The plant material is dried in the shade and ground into powder using a household blender for later use. Plant materials were dried in the shade and powdered by the home grinder.

Experimental research and field studies on cultivated and wild plants, including the collection of plant material, comply with relevant institutional, national, and international guidelines and legislation. This plant grows naturally in the different plains of Iran Natural Resources. The plants were collected to comply with relevant national policies and legislation of the Fars Agricultural and Natural Resources Research and Education Center (Fars, Iran). Permission was issued to collect the plant materials for this investigation. Agricultural and Natural Resources Research and Education Center confirmed all the source and batch number details. The qualitative chemical components of *Satureja* spp were considered using Fourier transform infrared (FT-IR) spectroscopy with FTIR spectrophotometer (Bruker, Germany)^14^.

### Fatty acids preparation and profiling

A modified and developed method of Folch containing chloroform: methanol: water (2:1:0.8, v/v/v) was used to extract and prepare total lipid^15^. Fatty acids methyl ester from whole lipid extract (1.0 g) were prepared with 5.0 mL of acidic methanol (methanol: sulfuric acid in 80:20 v/v) for 120 min at 80 °C. At that time, 5.0 mL of NaCl (0.7%) was added and vortexed for 5.0 min. Then, 5.0 mL of hexane was added and vortexed for 5.0 min, then centrifuged at 3000×*g* for 10 min. The fatty acid profile was analyzed using an Agilent gas chromatograph (Agilent 7890B GC 7955A MSD) coupled with a single quadrupole mass spectrometer and equipped with a fused silica capillary HP-5MS column (30 m × 0.25 mm; thickness 0.25 µm) according to previously published work^14^. The fatty acid was diluted in hexane, and 1.0 µL was injected into the column. The initial and maximum temperatures were 50 °C and 300 °C, respectively. The electron impact ionization mode was used for the mass spectrometric detection. Mass spectral was obtained from 40 to 650 Da at 70 eV electron ionization energy. The scanning time was 58 min. The split ratio was 1:50. The source and quadrupole were adjusted to 230 °C and 150 °C, respectively, while the interface was heated to 240 °C. The Oven temperature program was 60–280 °C as follows: 60 °C for 1.0 min, rise at 5 °C/min to 220 °C, and rise at three °C/min to 280°C^14^. The fatty acid components were determined by comparing the related peak retention times with those reported in the libraries (Wily 7n and NIST05a).

The lipid quality is evaluated by measuring total saturated fatty acids (SFA), monounsaturated fatty acids (MUFA), unsaturated fatty acids (UFA), polyunsaturated fatty acids (PUFA), omega-6/omega-3 (ω6/ω3) ratio, PUFA/SFA (P/S), atherogenicity index (AI), hypocholesterolemic index (HI), nutritional value index (NVI), thrombogenicity index (TI), health-promoting index (HPI), linoleic acid/linolenic acid (LA/ALA), fish lipid quality (FLQ), and unsaturation index (UI)^16^ (Table S1 in supplementary file).

### Total antioxidant capacity of *Satureja* fatty acid

The total antioxidant capacity was evaluated by mixing the *Satureja* fatty acid (0.02 mL equal to 20 mg) with 1.0 mL of ABTS radical solution (2.54 mM potassium persulfate plus seven mM ABTS) and monitoring light absorbance at 734 nm. The percentage of radical inhibition and the 50% inhibitory concentration (IC_50_) were calculated using the change in absorbance at 734 nm. A calibration graph was drawn based on Trolox (1.0 mg/mL) as a standard reference. The antioxidant potential of *Satureja* fatty acid was measured in milligrams of Trolox equivalents (TE) per gram^17^. All measurements were performed in triplicate.

### Lipase inhibition

The fatty acid was dissolved at 20 mg per mL (equal to 0.02 mL/mL) in tween-80 (0.3%). To determine the lipase (EC 3.1.1.3, from *Saccharomyces cerevisiae,* Sigma-Aldrich, St Louis, MO, USA) inhibitory activity, lipase (2.0 U/mL) was incubated with *Satureja* fatty acid and orlistat (0.03 mL, 0.03–0.6 mg/mL) for 30 min at 37 °C. p-nitrophenyl butyrate (pNPB, 0.03 mL, 10 mg/mL) was added to the mixture to start the reaction. For 30 min, light absorption at 405 nm was measured. The milligrams of orlistat equivalents per gram of samples were used to measure the anti-lipase activity. Using the change in absorbance, the inhibitory concentration that inhibits lipase by 50% (IC_50_) and the lipase inhibition percentage was computed^18^. In the presence of inhibitors, the Lineweaver–Burk plot was drawn based on 1/[pNPB] versus 1/velocity to study the lipase kinetics. The substrate and inhibitors were pNPB (0.0–10 mg/mL) and orlistat (0.3 mg/mL) or *Satureja* fatty acid (0.3 mg/mL), respectively. The quantitative pNPB breakdown per minute was determined using light absorbance monitoring at 405 nm. The lipase activity was shown by the slope of changes in light absorbance over time. The kinetic parameters of lipase inhibition were determined through the Lineweaver–Burk plot.

### Amylase inhibition

To determine the α-amylase (EC 3.2.1.1, from Bacillus sp., Sigma-Aldrich, St Louis, MO, USA) inhibitory activity of *Satureja* fatty acid, the solution of amylase (0.04 mL of 2.0 U/mL) was pre-incubated with inhibitors (0.04 mL of 0.03–0.60 mg/mL of acarbose or *Satureja* fatty acid) at 37 °C for 20 min in a 5.0 mL vials. After the incubation, starch solution (0.25 mL of 10 mg/mL) was added. The mixture was incubated at 37 °C for 30 min. In the end, 0.10 mL of iodine reagent was added, and light absorbance was recorded at 580 nm^14^. The anti-amylase activity was expressed in milligrams of acarbose equivalents per gram of samples using the calibration graph with the positive control (acarbose). The inhibitory concentration resulting in 50% amylase inhibition (IC_50_) was calculated based on the change in light absorbance^19^. In the presence of *Satureja* fatty acid (0.30 mg/mL), the Lineweaver–Burk plot was employed for amylase kinetic assay based on the 1/[starch] versus 1/velocity plot. The starch (0.0–10 mg/mL) was used as substrate. Acarbose (0.30 mg/mL) and *Satureja* fatty acid (0.30 mg/mL) were used as inhibitors. The rate of starch breakdown for 30 min was measured using light absorbance monitoring at 580 nm. Amylase activity is determined by the slope of light absorbance change (starch degradation) versus time. The kinetic parameters of amylase were determined using the Lineweaver–Burk plot^12^.

### Fluorescence and ultraviolet spectroscopy analysis

Using ultraviolet and fluorescence spectroscopy, the interactions of amylase and lipase with *Satureja* fatty acid were examined^20^. Briefly, 0.5 mL of inhibitor solutions were added to the 0.5 mL of amylase or lipase solutions (5.0 mg/mL). The mixtures were placed for 10 min at ambient temperature. The UV absorption spectra of the enzyme-inhibitor solution were recorded using an ultraviolet absorption spectrophotometer (UV1280, Shimadzu, Japan). A fluorescence spectrophotometer (Varian Cary Eclipse, Agilent, USA) was used to study the intrinsic fluorescence absorption of enzyme-inhibitor solutions at excitation wavelengths of 280 nm and emission wavelengths of 290 to 500 nm.

### Macrophage cell line culture

The hematopoietic mouse macrophage cell line (J774.A1, NCBI code, C483) was purchased from the cell bank of the Pasteur Institute of Iran. The macrophage cell line was grown in DMEM media supplemented with FBS (fetal bovine serum, 10%), glutamine (2.0 mM), streptomycin (0.10 mg/mL), and penicillin (100 U/mL). Cytotoxic effects of *Satureja* fatty acid on the macrophages were assessed using an MTT assay. Macrophage cells and *Satureja* fatty acid were added to tissue culture plates at concentrations ranging from 0.0 to 0.2 mg/mL. The supernatant was then mixed with the MTT solution (0.5 mg/mL), and the mixture was incubated for four hours at 37 °C. The MTT was replaced with DMSO to dissolve the formazan crystals. Light absorption at 492 nm was measured to evaluate cell viability. Macrophage cells were incubated with LPS (2.0 mg/mL) and non-cytotoxic concentrations of *Satureja* fatty acid to assess their inflammatory marker. The expression of NOX, NF-kB, NRF2, and hydrogen peroxide production were tested in the treated cells^21^.

### Hydrogen peroxide measurement

The hydrogen peroxide production was monitored using FOX (Ferrous oxidation-xylenol orange) reagent. The FOX solution contained xylenol orange (0.250 mM), ferrous ions (0.250 mM), and perchloric acid (110 mM). The culture medium (0.9 mL) and methanol (0.1 mL) were mixed, and the combination was incubated at room temperature for 30 min. After adding the FOX reagent (0.9 mL), the mixture was incubated for 30 min, and light absorbance at 560 nm was measured^22^.

### NOX activity

Sodium dodecyl sulfate (SDS) is used to lyse macrophage cells. The NOX activity is measured using a reaction solution containing dithiothreitol (1.0 mM), sodium phosphate (100 mM), and NADH (100 mM). The reaction began when 0.5 mL of the reaction solution was added to 0.5 mL of the cell extract. NADH breakdown is followed by the measurement of light absorbance at 345 nm. The decomposition of 0.001 mM NADH per minute is the unit of NOX activity^23^.

### Whole RNA obtaining and analysis of the real-time PCR

Total RNA is obtained using an RNA extraction kit (TRNX-plus, Cinagen, Tehran, Iran). First-strand DNA reagent (Fermentas, Hanover, MD) was used to synthesize first-strand cDNA from 0.001 mg of mRNA. A second-strand cDNA kit (Invitrogen, ThermoFisher Scientific) was used to synthesize second-strand cDNA using specific primers (Table S2 in supplementary file). The thermal program and the amplification cycles performed in the thermal cycler were completed (Line-Gene, Bioer Technology Co., Hangzhou, China). The threshold cycle (CT) was used to estimate the relative expression of the target genes. The Line-gene K program was used to compute the CT value for each gene^24^.

### Statistical evaluation

The data are presented as mean values plus standard deviations and derived from at least three research studies. One-way analysis of variance (ANOVA) and Tukey post-hoc tests were used to examine significant differences between treatments using the statistical package for the social sciences (SPSS, Abaus Concepts, Berkeley, CA) software. Relationships between the different samples and the association between essential oil profile and antioxidant, anti-amylase, and anti-lipase capacities were analyzed by principal component analysis (PCA). Minitab 18 statistical software was used to perform the PCA based on the correlation matrix.

## Results and discussion

### FTIR spectroscopy

**Figure 1:**
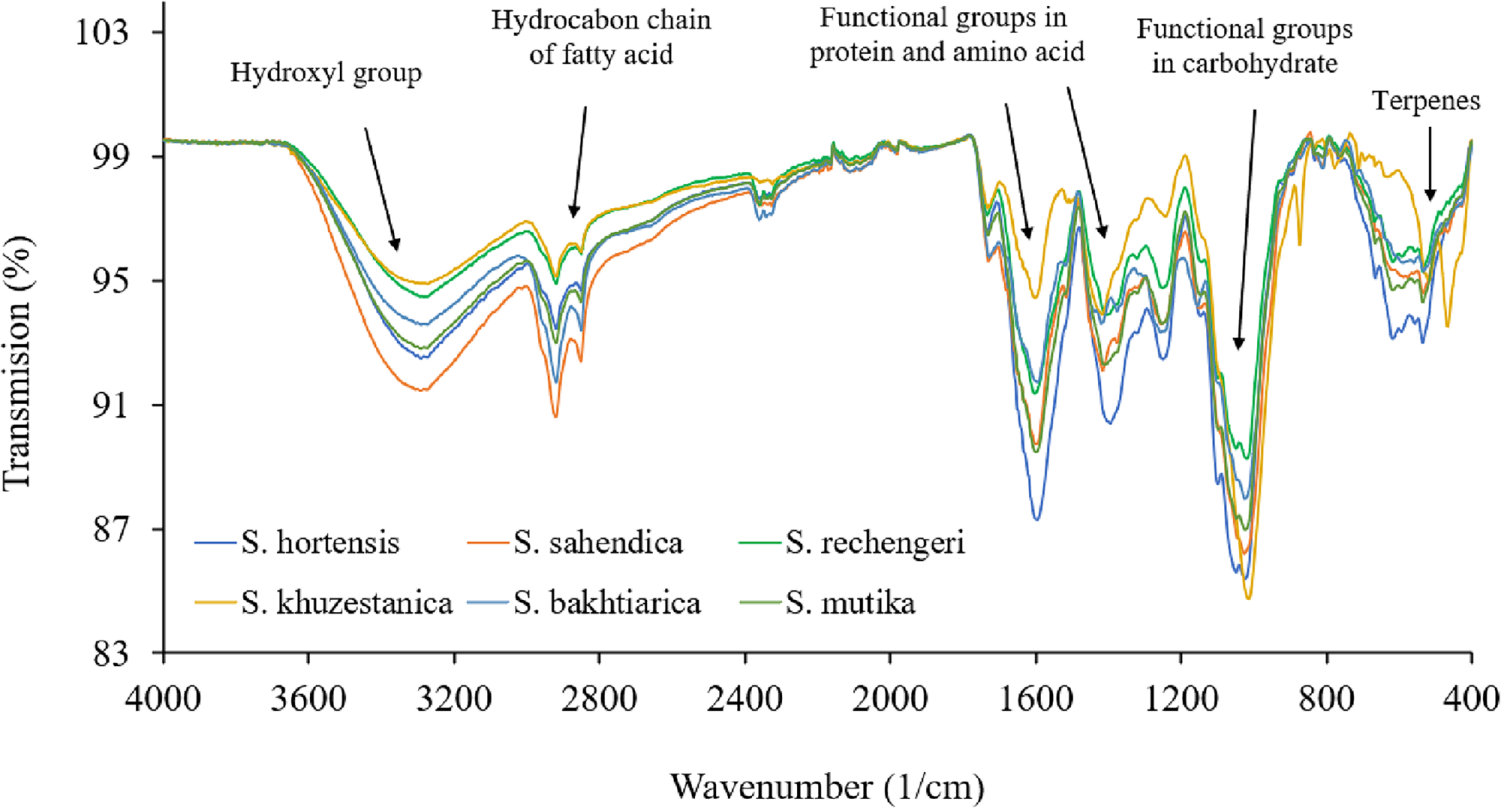
Furrier transforms-infrared spectra of *Satureja*.

**Figure 2:**
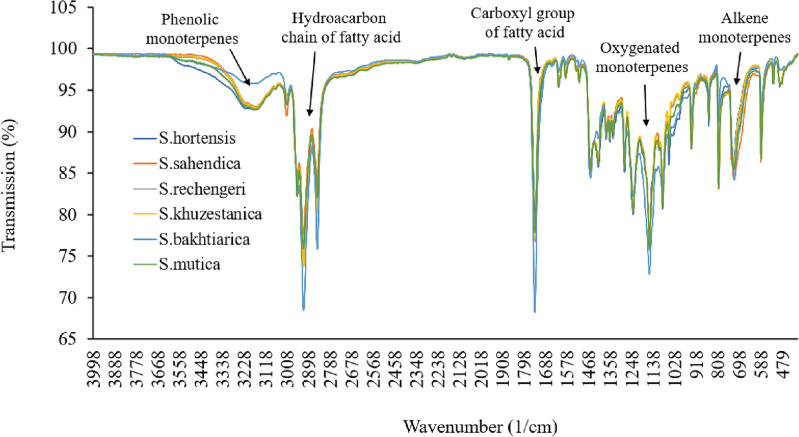
Furrier transforms-infrared spectra of *Satureja* fatty acid.

According to FT-IR analyses, *Satureja* had a high carbohydrate content, moderate protein and fatty acids, and low phenolic monoterpenes and monoterpenes (Fig. 1). The broad peak observed at 3800–3100 cm^−1^ indicated the presence of the OH functional group due to the stretching vibration of water or hydrogen in hydrogen bonds. Such peaks stated the existence of alcohol and phenyl groups. The bands at 2930–2900 cm^−1^ can be attributed to C–H vibrations, including CH, CH_2_, and CH_3_ stretching of fatty acids, hydrocarbon chain of amino acid, aliphatic chain, alkynes, and aldehydes. The peaks at 1750–1500 cm^−1^ were consigned to the C=O stretch, the C=C stretch of aromatic compounds, amide I and II, and amide II of the protein component. Stretching of carbohydrates C–O, C–C, and C–O–H are reflected in the peaks at 1150–100 cm^−1^. The bands at 1100–900 cm^−1^ resulted from C–O–H and =CH bending of carbohydrates. The 1000–800 cm^−1^ spectrum band can be assigned to C–H bending due to terpenes^25^. The Satureja fatty acid had a high level of fatty acid, especially unsaturated types, and low levels of monoterpenoids and monoterpenes (Fig. 2).

### Chemical composition of *Satureja* fatty acid

The main fatty acid components in the *Satureja* are stated in Table 1. The yield of fatty acid (oil) was averaged between 8.5 and 9.5%. The main components of the fatty acids were linolenic acid (23.58–37.32%), palmitic acid (10.65–15.08%), linoleic acid (8.31–13.39%), oleic acid (4.42–14.35%), stearic acid (2.75–8.77%) and palmitoleic acid (1.77–4.95%). Previous results (Table S3 in the supplemental file) suggested linolenic acid (48.56%), palmitic acid (16.25%), linoleic acid (14.59%), oleic acid (11.40%), and stearic acid (3.5%) as the main fatty acid components of *Satureja* ^26,27^, to some extent similar to these results especially in essential fatty acids, linolenic and linoleic acids. Given the lipid nutritional quality, omega-3 PUFA (23.58–37.31%), SFA (21.53–26.69%), omega-6 PUFA (10.80–16.14%), omega-9 MUFA (4.42–14.35%), and omega-7 MUFA (1.77–4.95%) make up the majority of fatty acids. *Satureja* had promising unsaturation index (120.77–164.26), PUFA/MUFA (2.08–6.40), hypocholesterolemic index (2.44–3.47), health-promoting index (2.03–2.42), PUFA/SFA (1.37–1.93), nutritive value index (0.52–1.71), MUFA/SFA (0.30–0.80) omega-6/omega-3 (0.33–0.65), atherogenicity index (0.41–0.49), linoleic acid/linolenic acid index (0.30–0.54), and thrombogenicity index (0.17–0.27) (Table 1). Limited work has been conducted on the elucidation of the fatty acid composition and lipid nutritional quality of Satureja and other Lamiaceae family. However, these rare reports suggested that the Lamiaceae family has an excellent balance of SFA (myristic, palmitic, stearic acids) and UFA (oleic, linolenic, and linoleic acids) and high content of PUFA (omega-3 and omega-6) and MUFA (omega-9 and omega-7) with promising lipid nutritional quality^26,27^. To treat and prevent hypertension, inflammation, obesity, anti-diabetes, and metabolic syndrome, *Satureja* fatty acid could be employed as a food ingredient and dietary supplement^28^.

**Table 1:** Fatty acid and monoterpenes composition (%) of *Satureja*.

Name	*S. hortensis*	*S. rechingeri*	*S. sahendica*	*S. bakhtiarica*	*S. khozestanica*	*S. mutika*
Thymyl methyl ether	0.73 ± 0.03b	0.50 ± 0.02c	1.96 ± 0.09a	1.59 ± 0.07	0.84 ± 0.04	1.06 ± 0.06
Caryophyllene	1.05 ± 0.05a	0.68 ± 0.03b	0.83 ± 0.04a	0.88 ± 0.04	0.91 ± 0.04	0.86 ± 0.04
Cuminaldehyde	0.62 ± 0.03b	1.40 ± 0.06a	0.78 ± 0.03b	0.31 ± 0.01	0.45 ± 0.02	0.94 ± 0.05
Thymol	2.87 ± 0.13c	2.79 ± 0.12c	7.27 ± 0.32a	3.93 ± 0.17	2.89 ± 0.13	4.31 ± 0.27
Carvacrol	10.64 ± 0.47b	18.86 ± 0.84a	10.54 ± 0.47b	17.94 ± 0.80	20.29 ± 0.90	13.35 ± 0.80
Decanoic acid (C10:0)	0.77 ± 0.03a	0.53 ± 0.02b	0.65 ± 0.03b	0.40 ± 0.02	0.99 ± 0.04	0.65 ± 0.03
Dodecanoic acid (C12:0)	1.66 ± 0.07a	1.65 ± 0.07a	1.56 ± 0.07a	1.30 ± 0.06	1.99 ± 0.09	1.62 ± 0.07
Tetradecanoic acid (C14:0)	1.86 ± 0.08b	2.70 ± 0.12a	2.23 ± 0.10a	2.50 ± 0.11	1.98 ± 0.09	2.26 ± 0.12
9-Tetradecenoic acid (C14:1n5)	1.60 ± 0.07a	0.88 ± 0.04b	1.77 ± 0.08a	1.11 ± 0.05	1.43 ± 0.06	1.42 ± 0.06
Hexadecanoic acid (C16:0)	15.08 ± 0.67a	11.04 ± 0.49b	12.03 ± 0.53b	10.65 ± 0.47	14.20 ± 0.63	12.72 ± 0.60
9-Hexadecenoic acid (C16:1n7)	1.77 ± 0.08b	4.95 ± 0.22a	3.17 ± 0.14a	1.87 ± 0.08	3.33 ± 0.15	3.30 ± 0.13
Octadecanoic acid (C18:0)	4.73 ± 0.21b	5.15 ± 0.23b	8.77 ± 0.39a	3.88 ± 0.17	2.75 ± 0.12	6.22 ± 0.45
9-Octadecenoic acid (C18:1n9)	4.42 ± 0.20c	5.02 ± 0.22c	9.64 ± 0.43b	14.35 ± 0.64	4.70 ± 0.21	6.36 ± 0.40
9,12-Octadecadienoic acid (C18:2n6)	11.21 ± 0.50b	13.39 ± 0.60a	11.19 ± 0.50b	8.31 ± 0.37	9.34 ± 0.42	11.93 ± 0.65
9,12,15-Octadecatrienoic acid (C18:3n3)	37.32 ± 1.66a	24.67 ± 1.10c	23.58 ± 1.05c	25.04 ± 1.11	28.77 ± 1.28	28.52 ± 1.40
6,9,12-Octadecatrienoic acid (C18:3n6)	1.37 ± 0.06b	2.75 ± 0.12a	1.80 ± 0.07b	2.50 ± 0.11	1.60 ± 0.07	1.97 ± 0.05
Eicosanoid acid (C20:0)	1.68 ± 0.07c	2.50 ± 0.11b	1.46 ± 0.06c	2.80 ± 0.12	3.27 ± 0.15	1.88 ± 0.04
Total	99.38 ± 4.42a	99.48 ± 4.42a	99.26 ± 4.41a	99.37 ± 4.42	99.77 ± 4.43	99.37 ± 4,30
Monoterpene	15.91 ± 0.71c	24.24 ± 1.08a	21.39 ± 0.95b	24.66 ± 1.10	25.38 ± 1.13	20.52 ± 1.20
Fatty acid	83.46 ± 3.71a	75.23 ± 3.34a	77.86 ± 3.46a	74.71 ± 3.32	74.38 ± 3.31	78.86 ± 3.45
Saturated fatty acid (SFA)	25.78 ± 1.15a	23.57 ± 1.05b	26.69 ± 1.19a	21.53 ± 0.96	25.21 ± 1.12	25.35 ± 1.20
Unsaturated fatty acid (UFA)	57.68 ± 2.56a	51.66 ± 2.30b	51.16 ± 2.27b	53.18 ± 2.36	49.17 ± 2.19	53.51 ± 2.45
UFA/SFA	2.23 ± 0.10a	2.19 ± 0.10a	1.92 ± 0.09a	2.47 ± 0.11	1.95 ± 0.09	2.12 ± 0.13
Monounsaturated fatty acid (MUFA)	7.78 ± 0.35e	10.85 ± 0.48c	14.58 ± 0.65b	17.33 ± 0.77	9.46 ± 0.42	11.08 ± 0.53
Polyunsaturated fatty acid (PUFA)	49.89 ± 2.22a	40.81 ± 1.81b	36.58 ± 1.63c	35.85 ± 1.59	39.71 ± 1.76	42.43 ± 2.20
PUFA/MUFA	6.40 ± 0.28a	3.76 ± 0.17c	2.51 ± 0.11c	2.08 ± 0.09	4.19 ± 0.19	4.23 ± .20
Omega-3	37.31 ± 1.66a	24.67 ± 1.10c	23.58 ± 1.05c	25.04 ± 1.11	28.70 ± 1.28	28.52 ± 1.50
Omega-5	1.60 ± 0.07a	0.88 ± 0.04b	1.77 ± 0.08a	1.11 ± 0.05	1.43 ± 0.06	1.42 ± 0.05
Omega-6	12.57 ± 0.56b	16.14 ± 0.72a	12.99 ± 0.58b	10.80 ± 0.48	10.94 ± 0.49	13.91 ± 0.70
Omega-7	1.77 ± 0.08b	4.95 ± 0.22a	3.17 ± 0.14a	1.87 ± 0.08	3.33 ± 0.15	3.30 ± 0.17
Omoga-9	4.42 ± 0.20c	5.02 ± 0.22c	9.64 ± 0.43b	14.35 ± 0.64	4.70 ± 0.21	6.36 ± 0.25
Omega-6/omega-3	0.33 ± 0.01a	0.65 ± 0.03a	0.55 ± 0.02a	0.43 ± 0.02	0.38 ± 0.02	0.51 ± 0.03
PUFA/SFA	1.93 ± 0.09a	1.73 ± 0.08a	1.37 ± 0.06a	1.66 ± 0.07	1.57 ± 0.07	1.68 ± 0.08
MUFA/SFA	0.30 ± 0.01b	0.46 ± 0.02b	0.54 ± 0.02b	0.80 ± 0.04	0.37 ± 0.02	0.44 ± 0.03
Linoleic acid/linolenic acid (LA/ALA)	0.30 ± 0.01a	0.54 ± 0.02a	0.47 ± 0.02a	0.33 ± 0.01	0.32 ± 0.01	0.44 ± 0.03
Health-promoting index (HPI)	2.38 ± 0.11a	2.19 ± 0.10a	2.27 ± 0.10a	2.42 ± 0.11	2.03 ± 0.09	2.29 ± 0.07
Unsaturation index (UI)	164.26 ± 7.3a	132.62 ± 5.8b	125.24 ± 5.5bc	120.77 ± 5.3	135.02 ± 6.0	140.71 ± 7.0
Hypocholesterolemic index (HI)	2.92 ± 0.13a	2.98 ± 0.13a	2.92 ± 0.13a	3.47 ± 0.15	2.44 ± 0.11	2.94 ± 0.12
Atherogenicity index (AI)	0.42 ± 0.02a	0.45 ± 0.02a	0.44 ± 0.02a	0.41 ± 0.02	0.49 ± 0.02	0.44 ± 0,03
Thrombogenicity index (TI)	0.17 ± 0.01a	0.21 ± 0.01a	0.27 ± 0.01a	0.18 ± 0.01	0.19 ± 0.01	0.22 ± 0.02
Nutritive value index (NVI)	0.60 ± 0.03a	0.92 ± 0.04a	1.53 ± 0.07a	1.71 ± 0.08	0.52 ± 0.02	1.02 ± 0.03

The values are expressed as means ± SD for three replicates (n = 3). Mean values with different letters within a row are significantly different (*p* < 0.05).

### Total antioxidant activity

*Satureja* fatty acid displayed antioxidant capacity with IC_50_ ranging from 354 to 428 µg/mL. *Satureja* fatty acid showed antioxidant capacity ranging from 410 to 475 mg Trolox/g. These values were equivalent to 37.27 to 43.18 mg Trolox equivalent per gram of *Satureja* dried powder (Table 2). These experimental results suggested that the *Satureja* fatty acid, which contains monoterpenoids (thymol and carvacrol) and polyunsaturated omega-3 and omega-6 fatty acids, display potent antioxidant activity. To some extent, these experimental results are similar to those of *Oliveria decumbens*, *Thymus kotschyanus*, *Trachyspermum ammi*, and *Zataria multiflora* fatty acids^19^. A growing body of evidence supports the therapeutic potential of omega-3 polyunsaturated fatty acids (n-3 PUFA), mainly docosahexaenoic (DHA) and eicosapentaenoic acid (EPA), on metabolic diseases based on their antioxidant and anti-inflammatory properties^29^. Some studies suggested that long-chain polyunsaturated fatty acids displayed antioxidant activity and thus can scavenge superoxide and reactive radicals in an unsaturation-dependent manner^30^. Furthermore, emerging evidence suggests that the antioxidant and anti-inflammatory properties of polyunsaturated omega-3 fatty acids seem to play a vital role in their effectiveness in patients with cardiovascular pathologies^31^.

**Table 2:** Total antioxidant activity of *Satureja* lipid hydrolysate.

Samples	IC_50_ (µg/mL)	TE (mg/g)
Trolox	165 ± 5.4e	970 ± 11a
*S. bakhtiarica*	380 ± 6.7c	458 ± 8.0c
*S. hortensis*	428 ± 7.6a	410 ± 6.5e
*S. khozestanica*	354 ± 8.0d	475 ± 7.7b
*S. mutika*	420 ± 8.7ab	417 ± 6.0de
*S. rechingeri*	388 ± 8.3c	444 ± 8.2c
*S. sahendica*	405 ± 9.0bc	425 ± 7.5d

Antioxidant capacity was expressed as the concentration needed for 50% (IC_50_) radical scavenging and Trolox equivalent (TE). The values are expressed as means ± SD for three replicate experiments (n = 3). The mean values with different letters within a column are significantly different (*p* < 0.05).

### Anti-lipase activity and kinetic analysis

**Figure 3:**
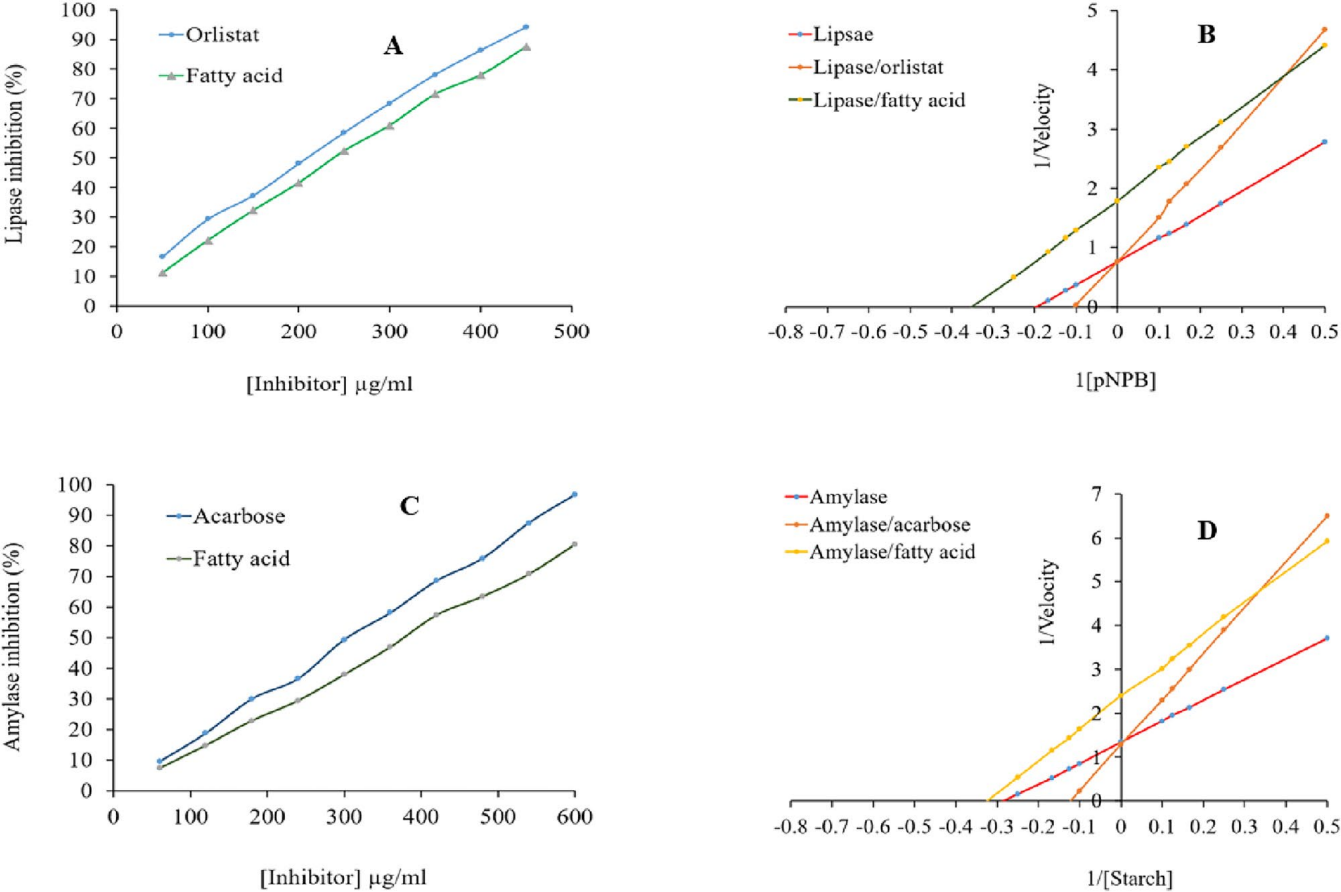
Amylase (**A**) and lipase (**C**) inhibition activity and Lineweaver–Burk plots of lipase (**B**) and amylase (**D**) inhibition in the presence of essential oil from *S. bakhtiarica*.

Triacylglycerols are broken down into fatty acids and glycerol by pancreatic lipase. Pancreatic lipase inhibitors can stop fatty acids from breaking down and entering the blood, which helps to lower lipid levels that can prevent obesity. Anti-obesity capacity was calculated based on lipase activity inhibition assay, and the results were reported as IC_50_ and orlistat equivalents per gram (Table 3 and Fig. 3). *Satureja* fatty acid displayed anti-lipase capacity with IC_50_ ranging from 270 to 293 µg/mL. *Satureja* fatty acid showed anti-lipase capacity ranging from 824 to 887 mg orlistat equivalent per gram oil. These values equal 75 to 80.6 mg orlistat per gram of *Satureja* dried powder (Table 3 and Fig. 3). Fatty acids are competitive inhibitors of lipases. Lipases hydrolyze ester bonds between fatty acids and glycerol; free fatty acids did not have an ester bond for hydrolysis. Fatty acids with a similar structure to monoacylglycerol can bind to the active site of lipase without any hydrolysis reaction and inhibit lipase^32^. Monoterpenes, monoterpenoids, omega-3/omega-6 fatty acids, or the combined effects of these compounds can all be associated with this lipase inhibitory capacity^33^.

**Table 3:** Anti-lipase capacity (IC_50_), orlistat equivalent (OE), and kinetic parameters (Km/Vmax, Km, and Vmax) of lipase in response to orlistat and fatty acid from *Satureja*.

Antidiabetic	IC_50_ (µg/mL)	OE (mg/g)	Km/Vmax	Vmax (µg/min)	Km (µg)
Lipase	–	–	3.91 ± 0.18c	1.29 ± 0.06a	5.08 ± 0.23b
Lipase + Orlistat	213 ± 5.0c	998 ± 15a	7.57 ± 0.33a	1.23 ± 0.05a	9.35 ± 0.45a
Lipase + *S. bakhtiarica*	280 ± 9.4a	835 ± 10 cd	5.163 ± 0.25b	0.55 ± 0.03b	2.84 ± 0.13c
Lipase + *S. hortensis*	270 ± 7.0b	887 ± 12ab	–	–	–
Lipase + S. khozestanica	275 ± 9.0ab	865 ± 13b	–	–	–
Lipase + *S. mutika*	278 ± 5.5ab	843 ± 12c	–	–	–
Lipase + *S. rechingeri*	288 ± 9.0a	830 ± 9.5 cd	–	–	–
Lipase + *S. sahendica*	293 ± 10a	824 ± 8.3d	–	–	–

The values are expressed as means ± SD for three replicates (n = 3). Mean values with different letters within a column are significantly different (*p* < 0.05).

The lipase K_m_/V_max_ increased while K_m_ and V_max_ declined in the presence of the *Satureja* fatty acid, according to the kinetic research based on the double reciprocal plot (Table 3 and Fig. 3). *Satureja* fatty acid inhibits lipase through competitive and non-competitive techniques. Monoterpenes, monoterpenoids, omega-3, and omega-6 fatty acids may all have a role in lipase inhibition. By raising the enzyme K_m_ and limiting triglyceride breakdown, small molecules can either occupy the triglyceride-binding site of lipase or act as triglyceride blockers. Large molecules can bind to allosteric regions in lipase, causing a conformational change, lowering lipase affinity for triglyceride, lowering Vmax, and slowing triglyceride breakdown. Conformational changes and activity inhibition occur when these bioactive compounds bind to active and allosteric sites^34^.

### Anti-amylase and kinetic analysis

Diabetes is characterized by hyperglycemia and glucose digestion system disturbances caused by an insulin section or action deficiency. In this case, amylase is an important enzyme that converts starch and oligosaccharide to glucose and affects blood glucose levels. Anti-diabetic activities were calculated based on amylase activity inhibition assay, and the results were reported as IC_50_ and acarbose equivalents per gram. *Satureja* fatty acid displayed anti-amylase capacity with IC_50_ ranging from 370 to 390 µg/mL. *Satureja* fatty acid showed anti-amylase capacity ranging from 752 to 790 mg acarbose equivalent per gram of fatty acid. These values equal 68.36 to 71.80 mg of acarbose equivalent per gram of *Satureja* dried powder (Table 4 and Fig. 3). These experimental results are similar to *Oliveria decumbens*, *Thymus kotschyanus*, *Trachyspermum ammi*, and *Zataria multiflora* fatty acids^19^. There is rare evidence that fatty acids, lipids, saponins, and lipophilic compounds found in fruits, vegetables, and mushrooms contribute to the in vitro α-glucosidase and α-amylase inhibition activities^35,36^. However, limited work has been conducted on the elucidation of the α-glucosidase and α-amylase inhibition activities of different fatty acids and lipids. Future studies are encouraged to investigate the individual inhibitors of cereal lipids and their inhibition mechanism. However, these rare results revealed that the presence of double bonds may play a crucial role in the anti-amylase activity of fatty acids. Additionally, unsaturated (oleic acid, linoleic acid, linolenic acid) and saturated (palmitic acid, stearic acid) fatty acids have been reported to have glucosidase and amylase inhibitory effects as competitive/uncompetitive inhibitors of starch-digestive enzymes, despite not structurally resembling carbohydrates^35,36^.

**Table 4:** Anti-amylase capacity (IC_50_), acarbose equivalent (AE), and the kinetic parameters (Km/Vmax, Km, and Vmax) of amylase in response to fatty acid *Satureja*.

Antidiabetic	IC_50_ (µg/mL)	AE (mg/g)	Km/Vmax	Vmax (µg/min)	Km (µg)
*Amylase*	–	–	4.705 ± 0.22c	0.743 ± 0.033a	3.498 ± 0.16c
*Amylase* + *Acarbose*	190 ± 6.0c	998 ± 15a	10.306 ± 0.47a	0.775 ± 0.034a	7.989 ± 0.37a
*Amylase* + *S. bakhtiarica*	370 ± 7.0b	790 ± 13b	7.211 ± 0.33b	0.427 ± 0.019c	3.079 ± 0.14c
*Amylase* + *S. hortensis*	390 ± 10a	752 ± 8.0c	–	–	–
*Amylase* + *S. khozestanica*	387 ± 8.0a	758 ± 10c	–	–	–
*Amylase* + *S. mutika*	380 ± 8.0ab	767 ± 12bc	–	–	–
*Amylase* + *S. rechingeri*	383 ± 7.6a	763 ± 11c	–	–	–
*Amylase* + *S. sahendica*	375 ± 9.0ab	770 ± 14b	–	–	–

The values are expressed as means ± SD for three replicates (n = 3). Mean values with different letters within a column are significantly different.

The double reciprocal plot suggests that in the presence of the fatty acid from *Satureja*, the amylase K_m_/V_max_ increased, and V_max_ decreased (p < 0.05). *Satureja* fatty acid did not have significant effects on the K_m_ of amylase. Accordingly, the *Satureja* fatty acid inhibits amylase un-competitively (Table 4 and Fig. 3). Amylase inhibition may be mediated by *Satureja* components, including phenolic compounds, monoterpenes, monoterpenoids, omega-3/-6 fatty acids, and unsaturated fatty acids. Small molecules may reside in the amylase active site or act as carbohydrate blockers by increasing enzyme K_m_ and limiting carbohydrate breakdown. Large molecules can bind to the allosteric region, changing the enzyme's conformation, decreasing the Vmax, and slowing the breakdown of polysaccharides. As soon as these bioactive compounds bind to the active and allosteric sites of amylase, conformational changes, and activity inhibition occur^37^.

### Ultraviolet and fluorescence spectroscopic analysis

**Figure 4:**
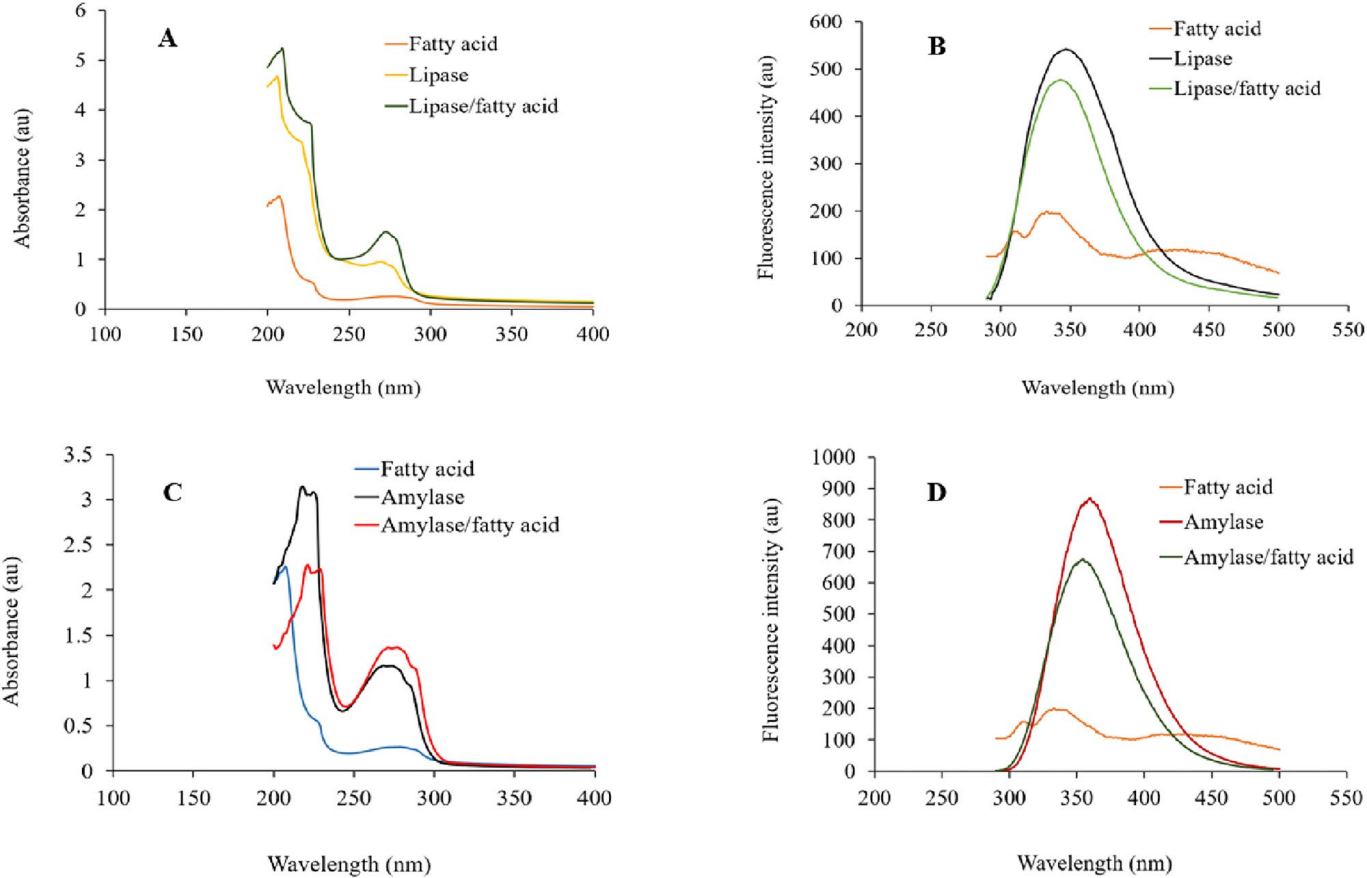
Ultraviolet absorbance (**A,C**) and Fluorescence intensity (**B,D**) of lipase (**A,B**) and amylase (**C,D**) in the presence of essential oil from *S. bakhtiarica*.

Ultraviolet–visible light absorption and fluorescence quenching analysis effectively reveal enzyme-inhibitor interactions. The ultraviolet spectrum of amylase (Fig. 4) and lipase (Fig. 4) at 270–280 nm increased in the presence of fatty acid. The fluorescence emission of amylase (Fig. 4) and lipase (Fig. 4) is reduced in the presence of the fatty acid. As a result, *Satureja* components can form non-covalent complexes with amylase and lipase, modify their conformation, and depict the aromatic group to Ultraviolet. Amylase and lipase ultraviolet light absorption increases, and fluorescence quenching establishes the non-covalent interaction between amylase and lipase and fatty acid components and reflects conformation changes^37^. These interactions depict the aromatic group to ultraviolet light leading to light absorption, on the other hand, producing a non-fluorescent enzyme-inhibitor compound, altering the enzyme microenvironment and architecture, and lowering the intrinsic fluorescence intensity^20^.

### Cytotoxicity of *Satureja* fatty acid

**Figure 5:**
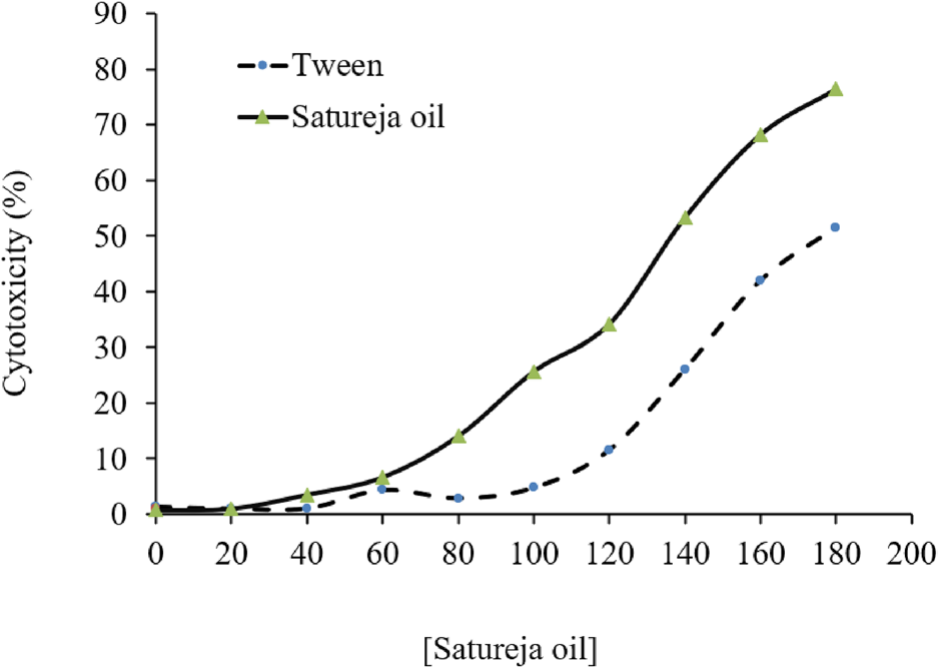
Cytotoxic activity of *Satureja* fatty acid on the viability of macrophages cell line.

The fatty acids from *Satureja* reduced the viability of macrophage cells in a concentration-dependent manner. The cytotoxicity rapidly increased at doses greater than 0.12 mg/mL (Fig. 5). Fatty acids are lipophilic substances that enter cells, disrupt organelle membranes, and cause cytotoxicity by penetrating and disrupting the cytoplasmic membrane. The fatty acid has a negligible effect on cytoplasm membranes and mitochondria at low concentrations. The presence of monoterpenes in the *Satureja* fatty acid can also destroy protein, DNA, and RNA, impair mitochondria, and cause cell death^38^. The cytotoxic effects of lipophilic compounds are ascribable to the lipophilic nature of the constituents that allow them to cross cell membranes, altering the phospholipid layers, increasing membrane fluidity, and leading to ions and cytoplasmic content leakage. The alteration of pH gradient reduced ATP production, and loss of mitochondrial potential are just a few consequences of disturbed cellular membranes^39^.

### Expression of oxidative biomarkers in cells treated with LPS

The levels of hydrogen peroxide, NOX, NF-kB, and NRF2 were low in untreated cells. Following LPS treatment, hydrogen peroxide, NOX, NF-kB, and NRF2 levels significantly rise (Table 5 and Figs. S1 and S2 in supplementary file). When LPS-stimulated cells were exposed to the non-toxic (0.04 mg/mL) concentration of the fatty acid of *Satureja*, the levels of hydrogen peroxide, NOX, and NF-kB activity were considerably decreased. At the same time, NRF was increased (Table 5 and Figs. S1 and S2 in supplementary file). These experimental results, to some extent, are comparable with the results of Najafi et al.^21^ on *Agastache foeniculum* fatty acids, Aminzadeh et al.^40^ on the *Zataria multiflora* essential oil, and the results of Obeidnejad et al.^22^ on *Satureja bakhtiarica* essential oil. It has been demonstrated that LPS causes superoxide and hydrogen peroxide synthesis and oxidative damage. Hydrogen peroxide and superoxide anion are mitogen-activated protein kinases (MAPK) stimulants. MAPKs induce NF-kB to increase the production of cytokines and inflammatory mediators. When LPS binds to the toll-like receptor 4 (TLR4), it triggers the translocation of NF-kB into the nuclei, which causes the production of several pro-inflammatory cytokines, such as tumor necrosis factor (TNF), interleukin-1 (IL-1), and IL-6^41^. Furthermore, it has been discovered that consuming extra omega-3 in the diet provides anti-inflammatory effects against several inflammatory disorders, including osteoarthritis and inflammatory bowel disease^42^. By inhibiting the TLR4/NF-kB signaling pathway, omega-3 can lessen the release of pulmonary inflammatory factors and oxidative stress in rats following acute lung damage^43^. Contrarily, omega-6 fatty acids are the building blocks for some pro-inflammatory mediators, such as prostaglandins and leukotrienes. Long-chain omega-6 fatty acids tend to produce pro-inflammatory eicosanoids, whereas omega-3 promotes the development of anti-inflammatory eicosanoids^44^. These results suggested that *Satureja* fatty acids with high omega-3 fatty acids can diminish hydrogen peroxide production and have anti-oxidative solid capabilities in stimulated macrophages.

**Table 5:** Modulatory effects of oil fraction (40 µg/mL) from *Satureja* on ROS production, NADH oxidase (NOX) activity, and related mRNA expression in LPS-stimulated macrophages.

Gene/protein	Control	LPS	LPS/*Satureja* oil
H_2_O_2_ (nM)	16.13 ± 2.25b	32.13 ± 2.44a	17.93 ± 1.54b
NOX activity	9.86 ± 1.15d	27.79 ± 2.25a	12.55 ± 1.20c
NOX22 mRNA	1.00 ± 0.20d	20.61 ± 1.96a	13.44 ± 1.10c
NOX40 mRNA	1.00 ± 0.20d	22.41 ± 1.45a	12.55 ± 1.13c
NOX47 mRNA	1.00 ± 0.20d	26.89 ± 1.75a	14.34 ± 1.10c
NOX67 mRNA	1.00 ± 0.20d	15.24 ± 1.65a	8.96 ± 1.0b
NF-kB mRNA	1.00 ± 0.20d	16.55 ± 1.33a	7.17 ± 0.70c
NRF2 mRNA	1.00 ± 0.20c	8.07 ± 0.88b	14.34 ± 1.27a

The values are expressed as means for three replicate experiments (n = 3). Mean values with different letters within a row are significantly different (p < 0.05).

Oxidative stimuli like LPS activate NRF2 and NF-kB. The NF-kB-IKB complex is the inactive variant of NF-kB in unstimulated cells. Inflammatory response and oxidative product are strengthened due to IKB release and NF-kB activation. Typically, the inactive form of NRF2 is the NRF2-Keap1 complex. In oxidative stress, Keap1 is released from NRF2 and activates NRF2. The NRF2 activation improves antioxidant defenses and superoxide neutralization coming on by NF-kB pathway activation. NRF2 is a key transcription factor that controls many aspects of cell homeostasis in response to oxidative stress. The target genes of NRF2 encode the antioxidant proteins, metabolic enzymes, transporters, redox balancing factors, and glutathione-conjugated coenzyme^45^. The NF-κB family regulates many genes involved in cellular processes, such as cell differentiation, proliferation, development, and apoptosis. The NRF2 and NF-κB pathways co-regulate cellular responses to oxidative stress and inflammation. In the cross-talk mechanism between NRF2 and NF-κB pathways, NRF2 and NF-κB compete for translocation to the nucleus, which depends on the relative amount of NRF2 and NF-κB. Furthermore, NRF2 is indirectly activated by anti-inflammatory compounds that suppress NF-κB activity; likewise, NF-κB is indirectly started by NRF2 inhibitors^46^. *Satureja* fatty acid had different modulatory effects on NF-kB and NRF2 because they significantly lowered NF-kB expression while raising NRF2 expression in the treated cells. These results point to their potential application as a preventive and therapeutic agent for oxidative damage brought on by inflammation.

### Principal component analysis

**Figure 6:**
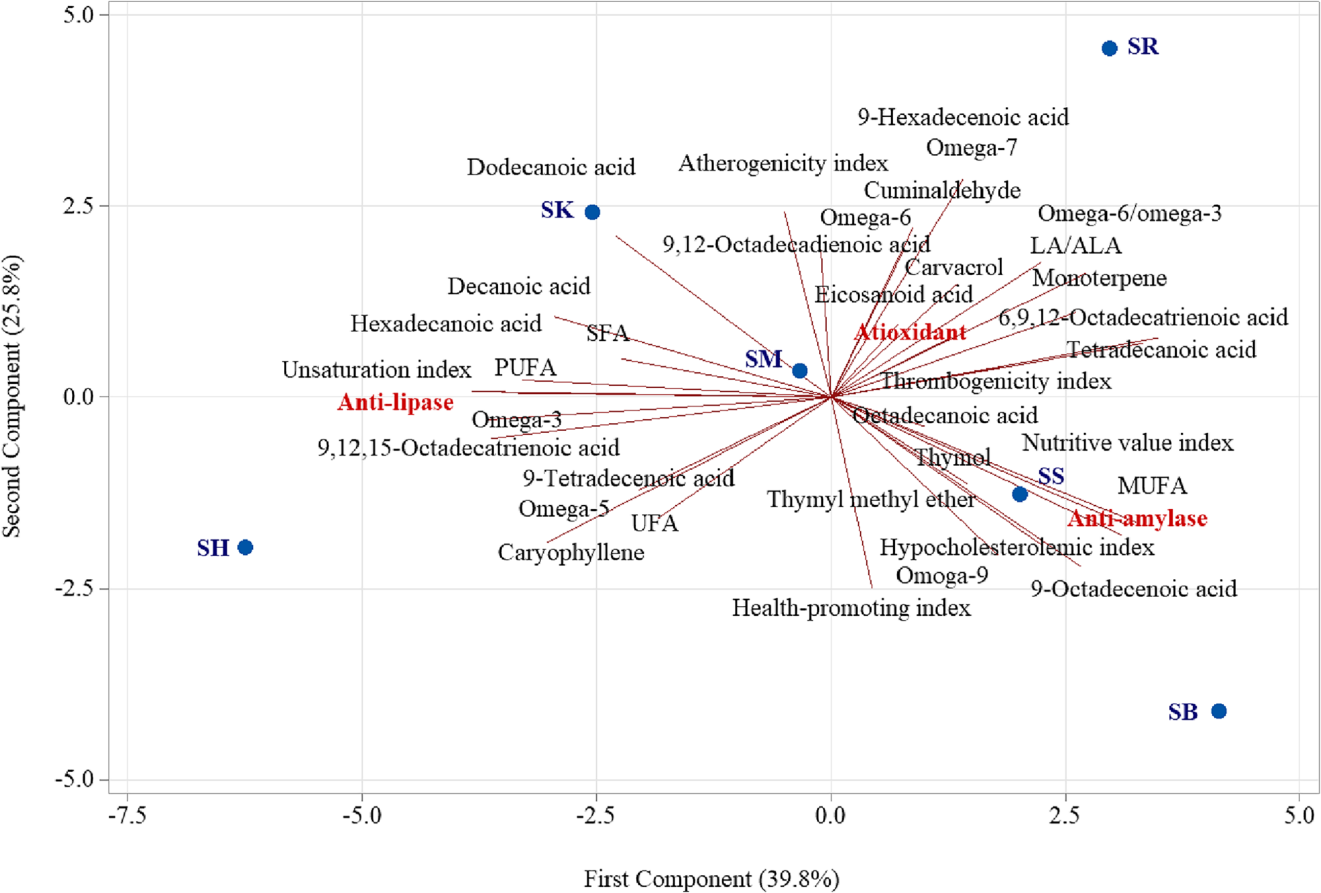
Principal component analysis (PCA) biplot illustrating the relationships among the fatty acid composition of *Satureja hortensis* (SH), *Satureja rechingeri* (SR), *Satureja sahendica* (SS), *Satureja bakhtiarica* (SB), *Satureja khuzistanica* (SK) and *Satureja mutika* (SM) and their antioxidant, anti-amylase and anti-lipase capacity.

PCA showed that the PC1 and PC2 accounted for 65.6% of the total variance of the changes, with the PC1 accounting for 39.8% and the PC2 for 25.8% (Fig. 6). Based on the PC1,* S. khuzistanica* (SK), *S. hortensis* (SH), and *S. mutika* (SM) are correlated with anti-lipase activity, and palmitic acid, linolenic acid, omega-3, unsaturation index, caryophyllene, decanoic acid, dodecanoic acid, 9-tetradecenoic acid, omega-5, atherogenicity index, and 9,12-octadecadienoic acid. Based on the PC1,* S. rechingeri* (SR), *S. sahendica* (SS), and *S. bakhtiarica* (SB) are correlated with anti-amylase and antioxidant capacity and tetradecanoic acid, linolenic acid, nutritive value index, omega-6/omega-3, 9-octadecenoic acid, omega-9, hypocholesterolemic index, thymyl methyl ether, thrombogenicity index, thymol, 9-hexadecenoic acid, omega-7, carvacrol, octadecanoic acid, cuminaldehyde, omega-6, eicosanoid acid, and health-promoting index^47^.

Based on the PC2,* S. hortensis* (SH),* S. sahendica* (SS), and *S. bakhtiarica* (SB) are correlated with anti-amylase and anti-lipase capacity and health-promoting index, 9-octadecenoic acid, omega-9, thymyl methyl ether, hypocholesterolemic index, caryophyllene, nutritive value index, 9-tetradecenoic acid, omega-5, thymol, 9,12,15-octadecatrienoic acid, omega-3, and octadecanoic acid. Based on the PC2,* S. sahendica* (SS), *S. khuzistanica* (SK), and *S. mutika* (SM) are strongly correlated with antioxidant capacity and 9-hexadecenoic acid, omega-7, atherogenicity index, cuminaldehyde, dodecanoic acid, omega-6, 9,12-octadecadienoic acid, carvacrol, decanoic acid, eicosanoid acid, tetradecanoic acid, 6,9,12-octadecatrienoic acid, thrombogenicity index, hexadecanoic acid, and unsaturation index^47^. Polyunsaturated fatty acids, especially omega-3 fatty acids, and hydrocarbon monoterpenes could inhibit lipase activity^48^. Likewise, phenolic monoterpenes like (thymol and carvacrol), omega-6 polyunsaturated fatty acids, and medium-chain fatty acids could inhibit amylase activity^19^.

## Conclusion

These findings showed that the main ingredients in *Satureja* fatty acids were linolenic acid, palmitic acid, linoleic acid, oleic acid, stearic acid, and palmitoleic acid. Omega-3 PUFA, SFA, omega-6 PUFA, omega-9 MUFA, and omega-7 MUFA comprise the majority of fatty acids from *Satureja*. *Satureja* fatty acid has a promising unsaturation index, hypocholesterolemic index, health-promoting index, nutritive value index, omega-6/omega-3, atherogenicity index, and thrombogenicity index. There is no report on the nutritional quality of *Satureja* fatty acid*.* However, after reviewing the literature and comparing the nutritional quality of fatty acids with other vegetable oils, we found that the nutritional quality of *Satureja* is healthier than that of common vegetable oils and is similar to that of meat and dairy products. *Satureja* fatty acid displayed strong antioxidant capacity, anti-lipase capacity, and anti-amylase capacity. LPS induced the expression of NOX, NRF2, and NF-kB and the synthesis of hydrogen peroxide in macrophage cells. In LPS-stimulated macrophages, *Satureja* fatty acid reduced NOX expression, hydrogen peroxide, and NF-kB expression and elevated NRF2. *Satureja* fatty acids have potent antioxidant, anti-amylase, anti-lipase, and anti-inflammatory activities. The common aspect between diabetes, obesity, and inflammation is oxidative stress. The mechanisms in lowering oxidative stress markers depended on down-regulating superoxide-producing enzymes at gene and protein levels. *Satureja* polyunsaturated omega-3 fatty acids could be recommended for healthy products combined with dietary therapy to treat obesity, diabetes, and oxidative stress. However, there is very rare evidence of fatty acids composition and quality and their functional activity on the in vitro and in vivo antioxidant and anti-inflammatory activities, as well as carbohydrate-digesting and lipid-digesting enzymes. Future studies are encouraged to investigate the molecular mechanism of oxidative stress and inflammatory response mitigation by omega-3 PUFA.

### Supplementary Information

Supplementary Information.

## Data Availability

The corresponding author will deliver the information assisting the findings of this study upon reasonable request.

## Abbreviations

LPS: Lipopolysaccharide
NOX: NADH oxide
NRF2: Nuclear respiratory factor-2
NF-kB: Nuclear factor-kappa B
PUFA: Polyunsaturated fatty acid
MUFA: Monounsaturated fatty acid
GC–MS: Gas chromatography-mass spectrometry
DMEM: Dulbecco modified eagle’s medium (DMEM)
FOX: Ferrous oxidation-xylenol orange
NADH: Nicotinamide adenine dinucleotide
MTT: 3-[4,5-Dimethylthiazole-2-yl]-2,5-diphenyltetrazolium bromide
DMSO: Dimethyl sulfoxide
FBS: Fetal bovine serum
MAPK: Mitogen-activated protein kinases
TLR2: Toll-like receptor 2
TLR4: Toll-like receptor 4
IkB: Inhibitor of kappa B
Keap1: Kelch-like ECH-associated protein 1
ABTS: 2,2'-Azino-bis(3-ethylbenzothiazoline-6-sulfonic acid)
