# Supplementary material for: Antioxidant, anti-amylase, anti-lipase, and efficiency of Satureja fatty acid on the anti-inflammatory parameters in lipopolysaccharide-stimulated macrophage through Nrf2/NF-kB/NADH oxidase pathway
Source: Sci Rep. 2024 May 31;14:12490. doi: 10.1038/s41598-024-63205-6 (PMC11143312; doi:10.1038/s41598-024-63205-6)
Supplement: Supplementary file 1 — Supplementary Information. [file 41598_2024_63205_MOESM1_ESM.doc]

**Antioxidant, anti-amylase, anti-lipase, and efficiency of *Satureja* fatty acid on the anti-inflammatory** **parameters in lipopolysaccharide-stimulated macrophage through Nrf2/NF-kB/NADH oxidase pathway**

Elham Obeidnejad^1^, Gholamreza Kavoosi^1^*, Mohammad Jamal Saharkhiz^2^

1. School of Agriculture, Department of Biotechnology, Shiraz University, Shiraz, Iran.

2. School of Agriculture, Department of Horticultural Science, Shiraz University, Shiraz, Iran

*Corresponding author: Gholamreza Kavoosi, School of Agriculture, Shiraz University, Shiraz, 7144113131, Iran. Tel/Fax: +98 (71) 332272805. Email: ghkavoosi@shirazu.ac.ir.

Short title: *Satureja* fatty acid

**Highlight**

1. Nutrient composition and lipase inhibitory of Satureja examined.

2. Satureja is composed of unsaturated fatty acids with high nutritional quality.

3. Satureja inhibits amylase and lipase with a non-competitive and uncompetitive strategy.

4. In LPS-stimulated macrophages, Satureja essential oil reduced NF-kB expression.

5. In LPS-stimulated macrophages, Satureja essential oil elevated NRF2 expression.

| **Table S1. Formulas for calculation lipid nutritional quality.** | |
| --- | --- |
| **Lipid nitritional index** | **Calculation Formula** |
| PUFA/SFA | PUFA/SFA |
| Index of atherogenicity | [C12:0 + (4 * C14:0) + C16:0] / UFA |
| Index of thrombogenicity | (C14:0 + C16:0 + C18:0)/[(0.5*(MUFA) + 0.5*(omega-6) + 3*(omega-3) +(omega-3/omega-6)] |
| Hypocholesterolemic/ hepercholestrolemic ratio | (C18:1n9) + PUFA) / (C12:0 + C14:0 + C16:0) |
| Hypocholesterolemic index | (C18:1n9+ C18:2n6+ C18:3n3+ C20:3+ C20:4n6+ C20:5n3+C22:4+ C22:6n3) / (C14:0+ C16:0) |
| Health-promoting index | UFA/[C12:0+ 4*(C14:0) + C16:0] |
| Linoleic acid/ linolenic acid ratio | C18:2 n-6/C18:3 n-3 |
| Fish lipid quality | 100 (C22:6 n-3 + C20:5 n-3)/SFA |
| EPA + DHA | C22:6 n-3 + C20:5 n-3 |
| Unsaturation index | 1*(% monoenoics) + 2*(% dienoics) + 3*(% trienoics) + 4*(% tetraenoics) + 5*(% pentaenoics) + 6*(% hexaenoics) |
| Nutritive value index (NVI) | (C 18:0 + C18:1)/C16:0 |
| Peroxidizability index (PI) | (Monoenoic acid × 0.025)1(dienoic acid×1.0)1(trienoic acid×2)1(tetraenoic acid×4)1(pentaenoic acid×6)1(hexaenoic acid×8). |
| Degree of unsaturation | MUFA + 2*(PUFA) |
| Long chain saturated fatty acid | (0.1*C16:0) + (0.5*c18:0) + (1*C20:0) + (1.5*C22:0) + (2*C24:0) |
|  |  |

| **Table S2. Primer from *Mus musculus* sequence used for real-time analysis.** | | | | |
| --- | --- | --- | --- | --- |
| **Genes** | **Genes** | **Accession No.** | **Sense sequence** | **Anti-sense sequence** |
| GAPDH | GAPDH | NM_008084 | 5′-CGGTGTGAACGGATTTGGC-3′ | 5′-TGAGTGGAGTCATACTGGAAC-3′ |
| NOX p22 | Cyba | NM_007806.3 | 5′- ATGGAGCGATGTGGACAG-3′ | 5′- ACCGACAACAGGAAGTGG-3′ |
| NOX p40 | Ncf4 | NM_008677.2 | 5′-CAACAAAGACTGGCTGGAG-3′ | 5′-CCGCAATGTCCTTGATGG-3′ |
| NOX p47 | Ncf1 | NM_001286037.1 | 5′- CAGAGATGACCGTGGCAACC-3′ | 5′- GGTTCACCTGCGTAGTTGGG-3′ |
| NOX p67 | Ncf2 | NM_010877.4 | 5′- CAGCCACAGTCAGCAGAG-3′ | 5′-GCACAAAGCCAAACAATACG-3′ |
| NF-kB | NF-kB | NM_08689 | 5′- GAAATTCCTGATCCAGACAAAAAC-3′ | 5′- ATCACTTCAATGGCCTCTGTGTAG-3′ |
| NRF2 | NRF2 | NM_010902.4 | 5′- CAGCGACAGAAGGACTATG-3′ | 5′- GCTCTATGCTGCTTAAATCA-3′ |
| Primer design, in the form of exon junction was carried out using Allele ID 7 software for the internal control and test genes from Mus musculus sequence. | | | | |

| **Table S3. Fatty acid composition of Satureja as reported in the literature**. | | | | | | | | | |
| --- | --- | --- | --- | --- | --- | --- | --- | --- | --- |
| **Name** | **S. hortensis^1^** | **S. boissieri^1^** | **S. hortensis^2^** | **S. thymbra^3^** | **S. cuneifolia^3^** | **S. amani^4^** | **S. montana^4^** | **S. illyrica^4^** | **Average** |
| Thymyl methyl ether | 0.00 | 0.00 | 0.00 | 0.00 | 0.00 | 0.00 | 0.00 | 0.00 | 0.00 |
| Caryophyllene | 2.26 | 4.12 | 0.00 | 0.00 | 0.00 | 0.00 | 0.00 | 0.00 | 0.80 |
| Cuminaldehyde | 0.00 | 0.00 | 0.00 | 0.00 | 0.00 | 0.00 | 0.00 | 0.00 | 0.00 |
| Thymol | 0.00 | 0.00 | 0.00 | 0.00 | 0.00 | 0.00 | 0.00 | 0.00 | 0.00 |
| Carvacrol | 0.00 | 0.00 | 0.00 | 0.00 | 0.00 | 0.00 | 0.00 | 0.00 | 0.00 |
| Decanoic acid (C10:0) | 0.00 | 0.00 | 0.00 | 0.00 | 0.00 | 0.00 | 0.00 | 0.00 | 0.00 |
| Dodecanoic acid (C12:0) | 0.00 | 0.00 | 0.00 | 0.10 | 0.00 | 0.00 | 0.00 | 0.00 | 0.01 |
| Tetradecanoic acid (C14:0) | 0.51 | 0.00 | 0.00 | 0.10 | 5.95 | 0.80 | 0.70 | 0.20 | 1.03 |
| 9-Tetradecenoic acid (C14:1n5) | 0.00 | 0.00 | 0.00 | 0.00 | 0.00 | 0.00 | 0.00 | 0.00 | 0.00 |
| pentadecanoic acid (C15:0) | 0.00 | 0.00 | 0.00 | 0.00 | 0.68 | 0.40 | 0.30 | 0.90 | 0.29 |
| Hexadecanoic acid (C16:0) | 17.95 | 22.94 | 3.61 | 11.40 | 58.82 | 5.10 | 4.80 | 5.40 | **16.25** |
| 9-Hexadecenoic acid (C16:1n7) | 1.69 | 0.00 | 0.39 | 0.10 | 1.19 | 0.50 | 0.40 | 1.00 | 0.66 |
| Octadecanoic acid (C18:0) | 0.00 | 0.00 | 1.52 | 14.10 | 3.06 | 2.20 | 1.90 | 5.20 | 3.50 |
| 9-Octadecenoic acid (C18:1n9) | 0.00 | 2.50 | 7.13 | 43.29 | 17.17 | 8.50 | 8.10 | 4.50 | **11.40** |
| 9,12-Octadecadienoic acid (C18:2n6) | 14.97 | 10.85 | 15.19 | 0.00 | 0.00 | 22.40 | 21.30 | 32.00 | **14.59** |
| 9,12,15-Octadecatrienoic acid (C18:3n3) | 56.53 | 52.89 | 66.24 | 30.20 | 10.71 | 59.65 | 62.00 | 50.28 | **48.56** |
| 6,9,12-Octadecatrienoic acid (C18:3n6) | 0.00 | 0.00 | 5.67 | 0.10 | 0.85 | 0.00 | 0.00 | 0.00 | 0.83 |
| Eicosanoid acid (C20:0) | 0.97 | 0.00 | 0.23 | 0.10 | 0.00 | 0.00 | 0.00 | 0.00 | 0.16 |
| Total | 94.88 | 93.30 | 99.98 | 99.49 | 98.43 | 99.55 | 99.50 | 99.48 | 98.08 |
| 1. Çaçan, E., Kokten, K., & Kilic, O. (2018). *Progress in Nutrition, 20*, 231-236.  2. Emre, İ., Kurşat, M., Yilmaz, Ö., & Erecevit, P. (2020). *Brazilian Journal of Biology*, *81*, 144-153.  3. Gören, A. C., Bilsel, G., Altun, M., & Satıl, F. (2003). *Zeitschrift für Naturforschung C*, *58*(7-8), 502-504.  4. Nurtazina, A.N., Khalmenova, Z.B., & Umbetova, A.K. (2016). *Chemistry of natural compounds*, *52*(4), 686-688. | | | | | | | | | |


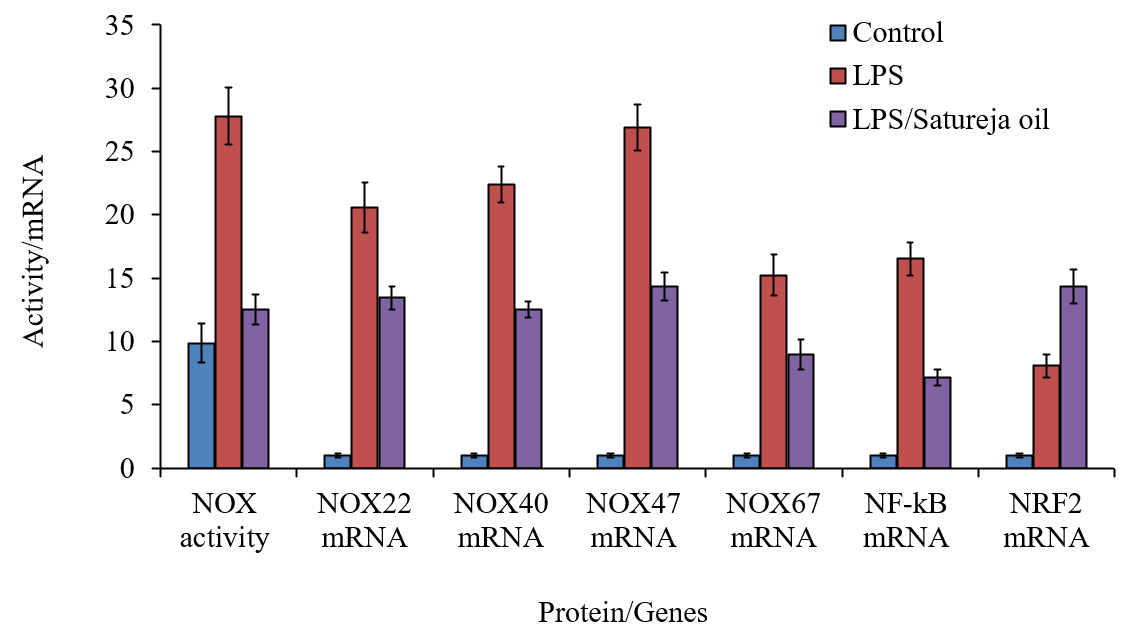


Figure S1. Modulatory effects of Satureja oil fraction (40 µg/mL) on NADH oxidase (NOX) activity and related mRNA expression in LPS-stimulated macrophages.


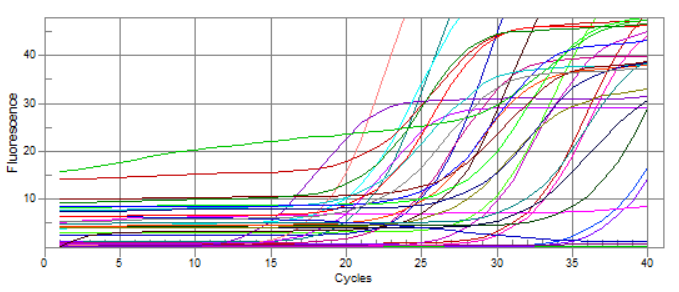


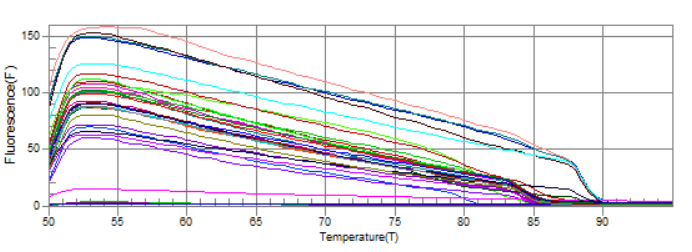


Figure S2. PCR profile of on NF-kB. NRF2, and NADH oxidase (NOX) mRNA expression in LPS-stimulated macrophages.
